# Screening the Budding Yeast Genome Reveals Unique Factors Affecting K2 Toxin Susceptibility

**DOI:** 10.1371/journal.pone.0050779

**Published:** 2012-12-05

**Authors:** Elena Servienė, Juliana Lukša, Irma Orentaitė, Denis L. J. Lafontaine, Jaunius Urbonavičius

**Affiliations:** 1 Laboratory of Genetics, Institute of Botany, Nature Research Centre, Vilnius, Lithuania; 2 Department of Chemistry and Bioengineering, Vilnius Gediminas Technical University, Vilnius, Lithuania; 3 Department of Biochemistry and Biotechnologies, Vytautas Magnus University, Kaunas, Lithuania; 4 Fonds de la Recherche Scientifique, Université Libre de Bruxelles, Charleroi-Gosselies, Belgium; 5 Center for Microscopy and Molecular Imaging, Académie Wallonie-Bruxelles, Charleroi-Gosselies, Belgium; University of Rome, Italy

## Abstract

**Background:**

Understanding how biotoxins kill cells is of prime importance in biomedicine and the food industry. The budding yeast (*S. cerevisiae*) killers serve as a convenient model to study the activity of biotoxins consistently supplying with significant insights into the basic mechanisms of virus-host cell interactions and toxin entry into eukaryotic target cells. K1 and K2 toxins are active at the cell wall, leading to the disruption of the plasma membrane and subsequent cell death by ion leakage. K28 toxin is active in the cell nucleus, blocking DNA synthesis and cell cycle progression, thereby triggering apoptosis. Genome-wide screens in the budding yeast *S. cerevisiae* identified several hundred effectors of K1 and K28 toxins. Surprisingly, no such screen had been performed for K2 toxin, the most frequent killer toxin among industrial budding yeasts.

**Principal Findings:**

We conducted several concurrent genome-wide screens in *S. cerevisiae* and identified 332 novel K2 toxin effectors. The effectors involved in K2 resistance and hypersensitivity largely map in distinct cellular pathways, including cell wall and plasma membrane structure/biogenesis and mitochondrial function for K2 resistance, and cell wall stress signaling and ion/pH homeostasis for K2 hypersensitivity. 70% of K2 effectors are different from those involved in K1 or K28 susceptibility.

**Significance:**

Our work demonstrates that despite the fact that K1 and K2 toxins share some aspects of their killing strategies, they largely rely on different sets of effectors. Since the vast majority of the host factors identified here is exclusively active towards K2, we conclude that cells have acquired a specific K2 toxin effectors set. Our work thus indicates that K1 and K2 have elaborated different biological pathways and provides a first step towards the detailed characterization of K2 mode of action.

## Introduction

Understanding the modes of action of biotoxins carries important implications both in fundamental and applied biology. The budding yeast (*S. cerevisiae*) killers serve as a convenient model to study the activity of biotoxins. The characterization of killer toxins has consistently provided significant insights into the basic mechanisms of self-defense and therefore immunity, in the mechanisms of virus-host cell interactions and toxin entry into eukaryotic target cells, and into biological processes as fundamental as RNA metabolism, protein maturation and secretion [1], [2]. In addition, studying killer yeasts and their toxins has many applications in important though diverse fields of human health (e.g. in the treatment of infections and in antifungal immunotherapy), of environmental biotechnology (e.g. in the development of antimycotic bioagents), and of the food industry (e.g. in pest control in the production of wine, beer or cheese) [3], [4], [5], [6].

Budding yeast produces four types of double-stranded RNA (dsRNA) virus-encoded killer toxins: K1, K2, K28, and the recently identified Klus [2], [7]. Killer K toxins are small proteins varying in size between 19 and 21.5 kDa. Toxin-producing yeasts are capable of killing both non-producers and producers belonging to a distinct type. Furthermore, they are immune to their own toxin, and to the strains belonging to the same killer group. Yeast killer toxins are translated into an inactive preprotoxin, which is imported into the secretory pathway where it is processed into a mature and cytotoxic α/β heterodimer, which is secreted into the medium [8], [9], [10]. Despite some similarities in their mode of production, killer K toxins have different biochemical properties (starting with their primary sequence) and drastically distinct modes of action. K28 toxin primarily acts in the nucleus of the host cell strongly interfering with gene expression, while K1 and K2 are mostly active at the cell wall where they ultimately disrupt the structural and/or functional integrity of the plasma membrane. The mode of action of Klus is not yet known [7].

The activity of K28 toxin depends on its retrograde passage through the secretory pathway, the endoplasmic reticulum (ER), and the nucleus, where it kills the host cells by irreversibly blocking DNA synthesis and by triggering G1/S arrest and apoptosis [11]. By contrast, K1 toxin disrupts the electrochemical ion gradient across the plasma membrane leading to uncontrolled leakage of K^+^ ions and small molecules from the cell [12], [13], [14]. The killing action of K1 toxin involves at least two events: first, a pH-dependent step during which the toxin binds to the cell wall; second, an energy-dependent step leading to the translocation and insertion of the toxin into the plasma membrane. The first step requires binding of the toxin to cell wall receptors, typically β-1,6-glucan [15]. Consistently, *kre* mutants, which are characterized by decreased amounts of cell wall β-1,6-glucan are resistant to K1 toxin [16]. During the second step, K1 toxin interacts with the plasma membrane receptor Kre1 [1] and disrupts the functional integrity of the plasma membrane either by inducing the formation of novel ion channels [17], or through the activation of Tok1 potassium channels [18].

The activity of K2 killer toxin was studied less extensively by comparison to K1 and K28, and it was generally assumed that K1 and K2 toxins act in a similar fashion [4], [9], [19]. However, there are several lines of evidence suggesting that the situation is more complex. Firstly, K1- and K2 toxin-producing strains are able to kill each other, whereas they are immune to their own toxin [19], [20]. This is *prima facie* evidence that K1 and K2 are functionally distinct. Secondly, K1 and K2 toxins are biochemically distinct: they differ in primary sequence, preprotoxin organization, molecular weight, isoelectric point, and killing pH optimum [19], [21], [22]. Thirdly, numerous budding yeast mutants have distinctive patterns of sensitivity/resistance towards K1 or K2 [23]. For example, Δ*kre2* cells lacking an α-1,2-mannosyltransferase are sensitive to K2 but are resistant to K1 [24], [25]. Interestingly, *KRE2* complements this phenotype not only in intact yeast cells but also in spheroplasts, which is a good indication that this resistance determinant to killer K toxin resides within the plasma membrane rather than the cell wall [26]. These differences between K1 and K2 toxins clearly call for further investigation. Genome-wide screens were performed to identify genes involved in the sensitivity/resistance towards the K1 and K28 killer toxins, revealing around 300–400 gene products for each toxin [27], [28]. Prior to this work, no such screening had been performed for K2 toxin, despite the fact that it is prevalent in the food industry.

Here, we have performed multiple genome-wide screens in *S. cerevisiae* following complementary experimental approaches in order to identify, with a high degree of confidence, novel cellular host factors involved in K2 toxin susceptibility. Our screens identified 332 gene products and demonstrate that those implicated in resistance to K2 toxin are involved in biological processes, or encode cellular components, markedly different from those implicated in hypersensitivity. Genes involved in resistance are directly connected to cell wall structure/biogenesis, including the formation of putative toxin receptors, and in mitochondrial function, while genes involved in hypersensitivity encode products active in osmosensory signaling and ion transport. Importantly, most genes identified in our screens (∼70%) had not previously been linked to the biology of K1 and K28 killer toxins. This demonstrates the existence of a set of specific host factors controlling the sensitivity/resistance of cells towards K2 killer toxin, thereby providing initial insights into its specific mode of action.

## Results

### Screening the Yeast Genome for Altered Resistance to K2 Killer Toxin

**Figure 1:**
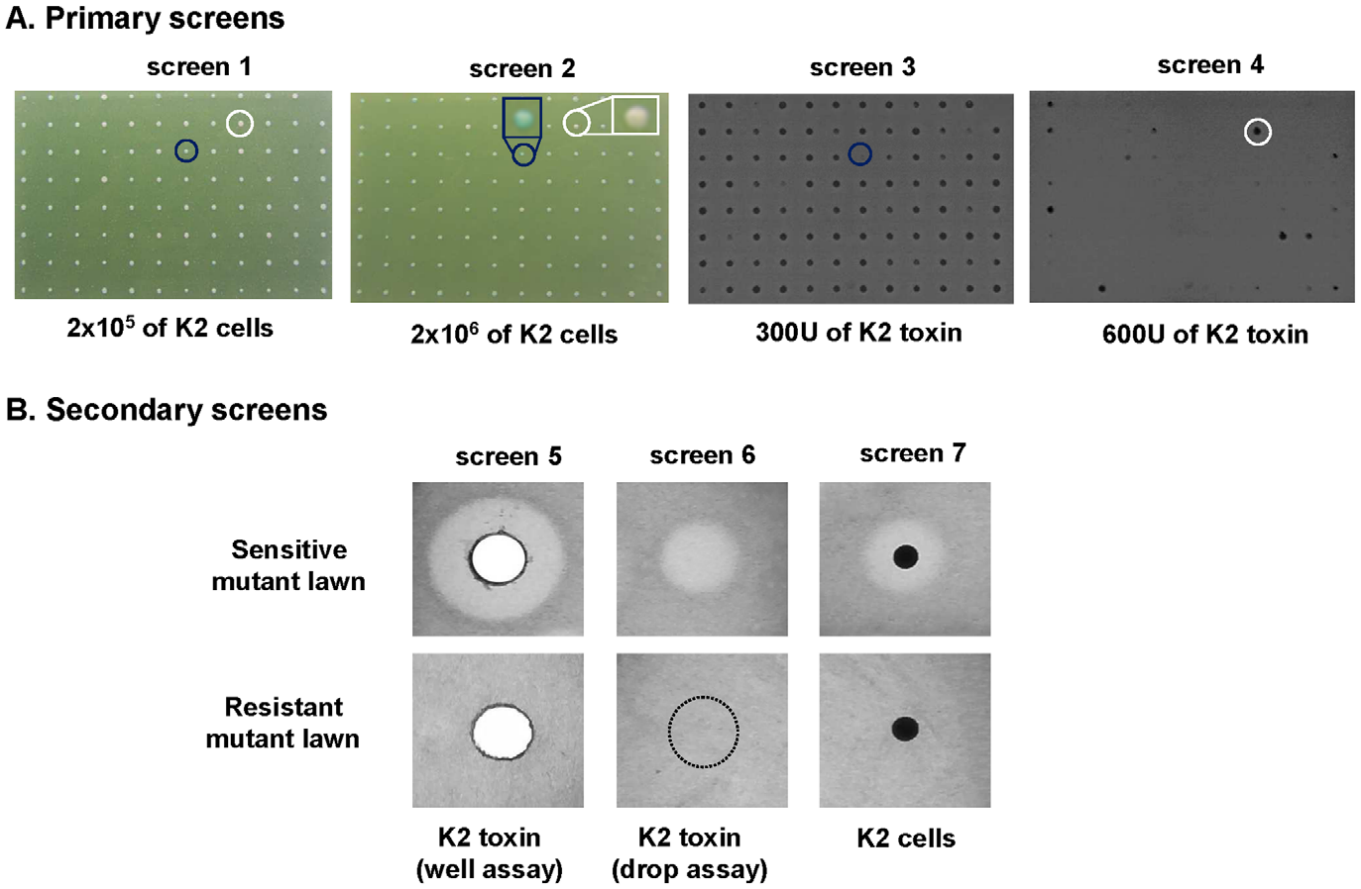
Setting up killer K2 toxin genome-wide screens. A. Primary screens. The yeast knockout library was arrayed on agar plates containing either 2×10^5^ (screen 1) or 2×10^6^ (screen 2) K2 toxin-producing cells or 300 U (screen 3) or 600 U (screen 4) of K2 toxin preparation. Cells were grown for 2 days at 25°C. A vital blue stain was used to ease the detection of dead cells. The plates were imaged with a digital color camera. Typically, small cyan colonies are hypersensitive to K2 toxin (see one example circled in blue), while large white colonies are hyper resistant (circled in white). Note that in the screens 3 and 4, the plates were imaged in grayscale only allowing scoring for the size of the colonies that appear in dark grey. B. Secondary screens: agar diffusion assays. Candidates identified in the primary screens were seeded inside the agar layer of test plates and either overlaid with purified K2 toxin (in a “punched-well” in the agar plate, or on the surface of the plate, in screens 5 and 6, respectively) or with K2 toxin-producing cells (screen 7). In screens No. 5–7, the test plates were imaged in gray scale. The mutant cells, inoculated inside the agar, appear as a light gray background whereas the “halo”, representing the area where cell growth was inhibited by the toxin, is transparent. In screen No. 7, a colony of K2-producing cells was deposited on the surface of the plate and appears in black. The primary screens were performed each once only. The secondary screens were each repeated three times.

To identify new gene products affecting the sensitivity/resistance of yeast cells towards the K2 killer toxin, we developed a novel screening procedure (see Figure 1). The rationale was to combine and integrate several approaches in order to improve the depth and robustness of the screening; i.e. to increase the number of candidates identified and to decrease the rate of false positives. We performed four individual primary screens on the haploid yeast knock out library consisting of 4774 strains, each carrying a deletion of a nonessential gene (Figure 1A). In screens 1 and 2, the library was replica plated on medium inoculated with K2 toxin-producing cells. In screens 3 and 4, the plates were directly prepared with purified K2 toxin. In both approaches, two concentrations of either K2 toxin-producing cells or K2 toxin were used. The size of the colonies was scored. This led to the identification of several hundreds of candidate genes involved in K2 resistance/hypersensitivity. Each candidate was then carefully validated in three independent secondary screens (see Figure 1B, screens 5–7). Here, each candidate strain was inoculated into the agar of test plates and overlaid either with K2 toxin (in screens 5 and 6) or with K2 toxin-producing cells (in screen 7). K2 toxin was either placed in a “punched-well” in the agar plate (screen 5) or simply deposited on the plate surface itself (screen 6). The size of the “halo”, revealing the growth inhibition induced by the toxin, was scored. The four primary screens were each performed once while the secondary screens were each repeated three times. Altogether, we identified 332 gene products whose absence either led to increased or decreased resistance to K2 toxin (see Tables S1 and S2). Note that the scoring categories, representing the whole range of sensitivity and resistance towards K2 toxin observed in our work, are shown in Figure S1. Out of 332 genes mutation in 205 caused marked (117 genes) or weak (88 genes) K2 resistance, whereas mutation in 127 led to K2 hypersensitivity (93 mutants with a marked and 34 with a weak phenotype).

### Genes Involved in Increased Resistance and Hypersensitivity to K2 are Distinctly Different

**Figure 2:**
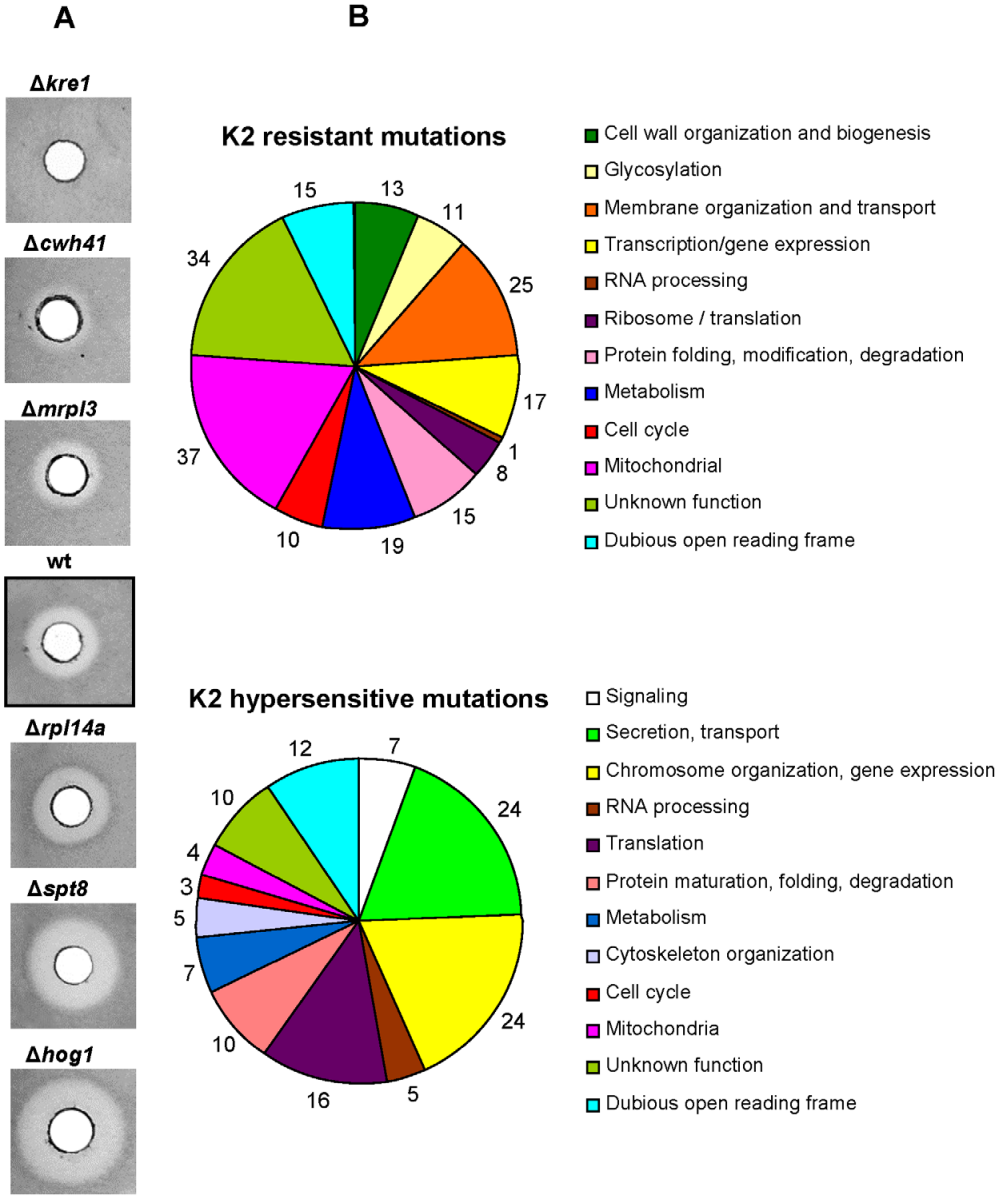
Distribution of cellular processes and cellular components involved in K2 resistance and hypersensitivity. A. Agar diffusion assays. Representative examples of “halo” observed for strains with different degree of sensitivity/resistance towards K2 toxin (see Figure 1B, screen 5, for details). An isogenic control (wt, strain BY4741) is provided in the center. Strains Δ*kre1*, Δ*chw41,* and Δ*mrpl3* are resistant (show a smaller “halo” than wild-type or no “halo”). Strains Δ*rpl14a*, Δ*spt8*, and Δ*hog1* are hypersensitive (show a larger “halo” than wild-type). B. Distribution of cellular pathways and cellular components, according to Gene Ontology, associated with the 205 genes associated with K2 resistance and 127 with K2 hypersensitivity. The number of genes identified in each class is indicated.

A full range of sensitivity/resistance towards K2 toxin among the mutants identified in our screens is illustrated in Figure 2A (the isogenic wild-type showing a “reference” halo in the center). Manual gene function assignment indicated that the genes involved in increased resistance and hypersensitivity to K2 toxin are involved in strikingly different biological processes or encode distinct cellular components (Figure 2B). Many genes associated with increased resistance to K2 are involved in cell wall organization and biogenesis (13 genes), membrane organization and transport (25), and glycosylation (11). In addition, and quite surprisingly, a large fraction of genes (37) are associated with mitochondrial function (see Discussion). On the other hand, many genes involved in K2 hypersensitivity have known functions in secretion and transport (24 genes), and altered gene expression (including functions in chromosome organization for 24 genes, and translation for 16 genes). Importantly, our screens allowed assigning a function to 44 genes (34 involved in K2 resistance; 10 in hypersensitivity) for which no function had previously been ascribed (see detailed list in the Tables S1 and S2).

**Figure 3:**
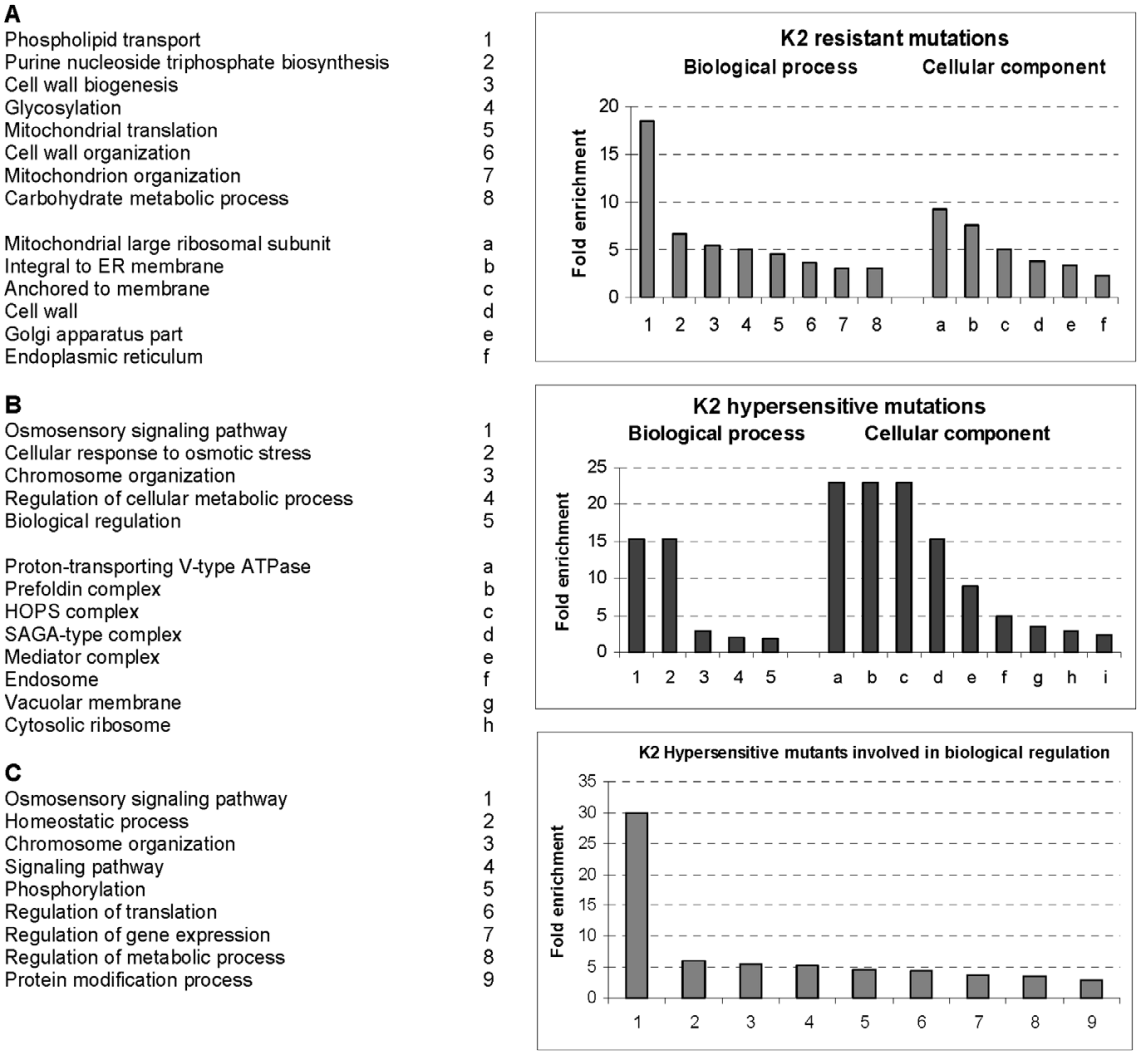
Statistically enriched gene ontology terms among putative K2 effectors. Fold enrichment (F.E.) was calculated by dividing the frequency of specific gene cluster to the total frequency for each GO term, according to the data highlighted in Table S3. Biological processes (1 to 9) and cellular components (a to h) are listed in each panel. A. Resistance. B. Hypersensitivity. C. Deconvolution of the genes involved in biological regulation shown in panel B.

To get further insights into the cellular activities involved in the biology of K2 killer toxin, we calculated the enrichment of ‘biological process’ and ‘cellular component’ gene ontology (GO) terms associated with pronounced K2 resistant and sensitive phenotypes (see Materials and Methods). We identified 117 GO terms that were statistically enriched (Benjamini and Hochberg False Discovery Rate corrected, with p<0.05 significance level) in our hit list (Table S3). Figure 3 illustrates the fold enrichment (F.E.) of the major GO terms associated with K2 susceptibility. Consistent with the results described above, mutations leading to increased resistance and hypersensitivity largely mapped in genes involved in distinct biological processes, or encoding distinct cellular components. Many mutations leading to resistance to K2 mapped in genes involved in phospholipid transport (F.E. of 18.5), as well as in cell wall integrity (biogenesis and organization, F.E. of 5.5 and 3.7, respectively). Genes involved in mitochondrial structure or function were again well represented (mitochondrial translation, mitochondrial organization, mitochondrial large ribosomal subunit, with respective F.E. of 4.6, 3.1 and 9.2) (Figure 3A). Mutations leading to increased sensitivity to K2 mapped in genes involved in osmosensory signaling and cellular response to osmotic stress (F.E. of ∼15 for each), as well as in genes encoding cellular components such as proton-transporting V-type ATPases, prefoldin and HOPS complexes (F.E. of 23.0), and transcription-related complexes such as SAGA and the mediator (F.E. 15.3 and 8.5 respectively) (Figure 3B). Genes concerned with biological regulation and leading to hypersensitivity were further deconvoluted (Figure 3C); the most represented GO term (F.E. of 30.0) was again found to correspond to osmosensory signaling.

The genome-wide screening thus revealed a clear dichotomy in the functions of the genes involved in resistance and hypersensitivity towards K2 killer toxin. Gene products involved in K2 resistance are mostly involved in cell wall and membrane structure/biogenesis, as well as in mitochondrial function, whereas genes whose deletion conferred K2 hypersensitivity are mostly connected to osmosensory signaling, homeostasis (ion transport and pH), and chromosome organization/gene expression.

### Gene Products Involved in K2 Biology are Physically and Functionally Highly Interconnected *in vivo*

**Figure 4:**
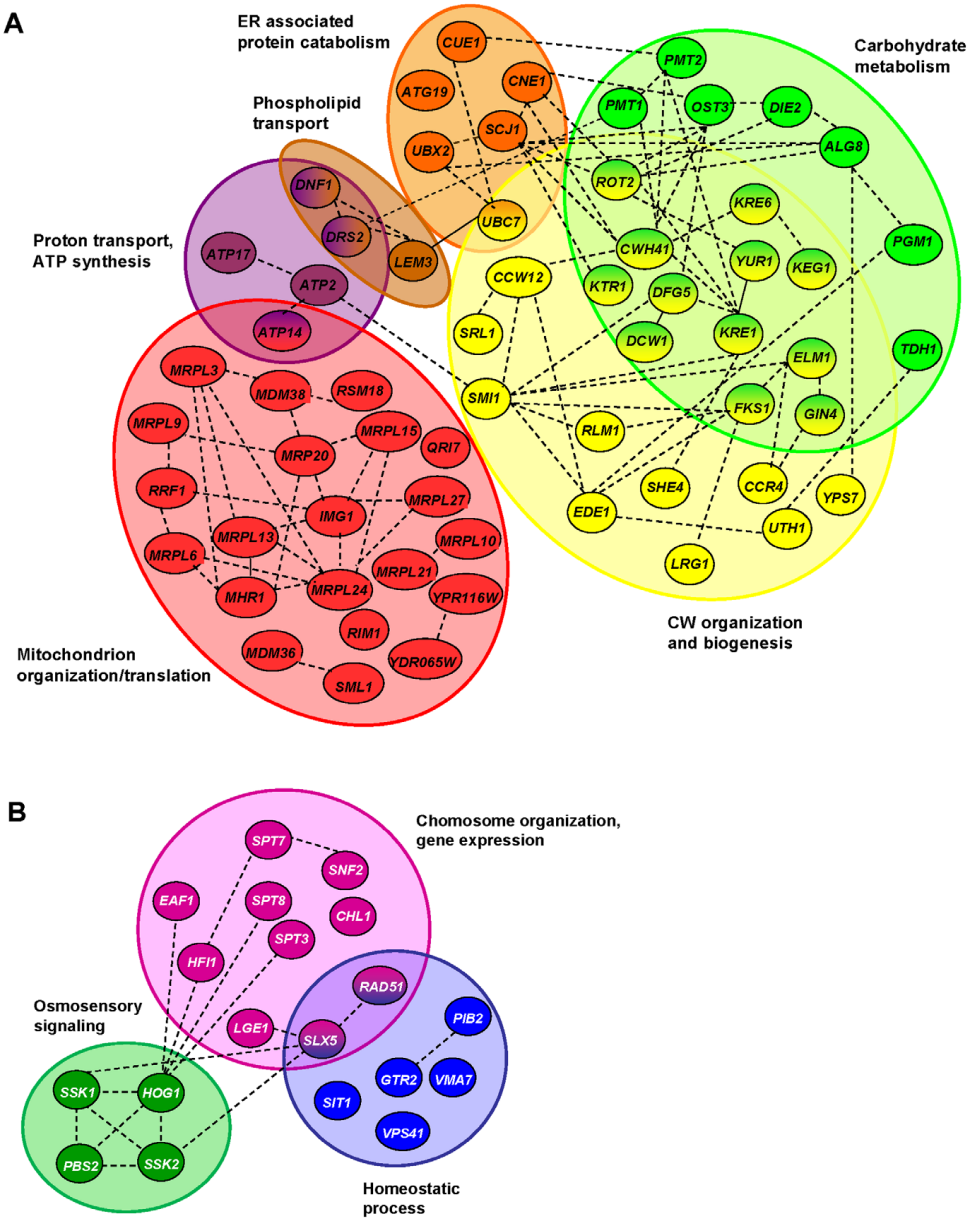
Gene products involved in K2 susceptibility are highly interconnected within cell. Physico-functional networks were established with STRING (see Materials and Methods and Figure S2). Gene products are depicted as color-coded nodes, according to cellular processes, and are connected by edges. Color coding is as follows: red, mitochondrion organization & translation; deep purple, proton transport & ATP synthesis; brown, phospholipid transport; orange, ER associated protein catabolism; yellow, cell wall organization & biogenesis; light green, carbohydrate metabolism; dark green, osmosensory signaling; light purple, chromosome organization & gene expression; blue, homeostatic process. Some nodes are connected through intermediates, which were not all represented here for simplification (see Figure S2 for details). A. Mutations leading to increased resistance. B. Mutations leading to increased hypersensitivity.

At this stage, we were interested in finding out whether the gene products identified in our genome-wide screens interact physically and/or functionally inside the cell. For this, we built two interaction networks, based on high throughput datasets available in the literature, one for gene products involved in increased resistance, and one for gene products involved in hypersensitivity. These networks demonstrated that the gene products involved in K2 biology are indeed highly interconnected (see Figure 4 and Figure S2).

As far as resistance to K2 is concerned, a sub-network of 23 gene products involved in cell wall organization and/or biogenesis (yellow nodes) was evident. Out of these, 12 gene products are also participating in carbohydrate metabolism (green nodes). Typically, these proteins are involved in the synthesis of structural components of the cell wall (e.g. Kre6, Cwh41, Yur1, Fks1), including a putative K2 toxin receptor Kre1. Another important group consists of 22 highly interconnected gene products directly involved in mitochondrial structure and function (red nodes). The remaining genes are connected to ER-associated protein catabolism (*CUE1*, *CNE1*, *SCJ1*, *UBX2*, *ATG19*, *QRI8*), proton transport/ATP synthesis (*ATP2*, *ATP14*, *ATP17*, *DNF1*, *DRS*2), and phospholipid transport (*DNF1*, *DRS*2, *LEM3*).

Amongst the mutants conferring a hypersensitive phenotype to K2 toxin (Figure 4B and Figure S2B), we noted a small cluster of four gene products involved in osmosensory signaling (dark green nodes: Hog1, Ssk1, Ssk2 and Pbs2). Also, seven gene products are participating in homeostatic processes (blue nodes: Gtr2, Pib2, Rad51, Sit1, Slx5, Vps41, Vma7). In addition, a group of ten gene products are linked to chromosome organization (pink nodes), including three subunits of the SAGA complex (Spt3, Spt7 and Spt8), and the SAGA adaptor protein Hfi1. Importantly, many of the genes identified are specific to K2 toxin; i.e. they have not been related to K1 or K28 toxin biology (see Discussion).

### K2 Toxin Binding Properties of Mutants with Altered K2 Resistance

**Figure 5:**
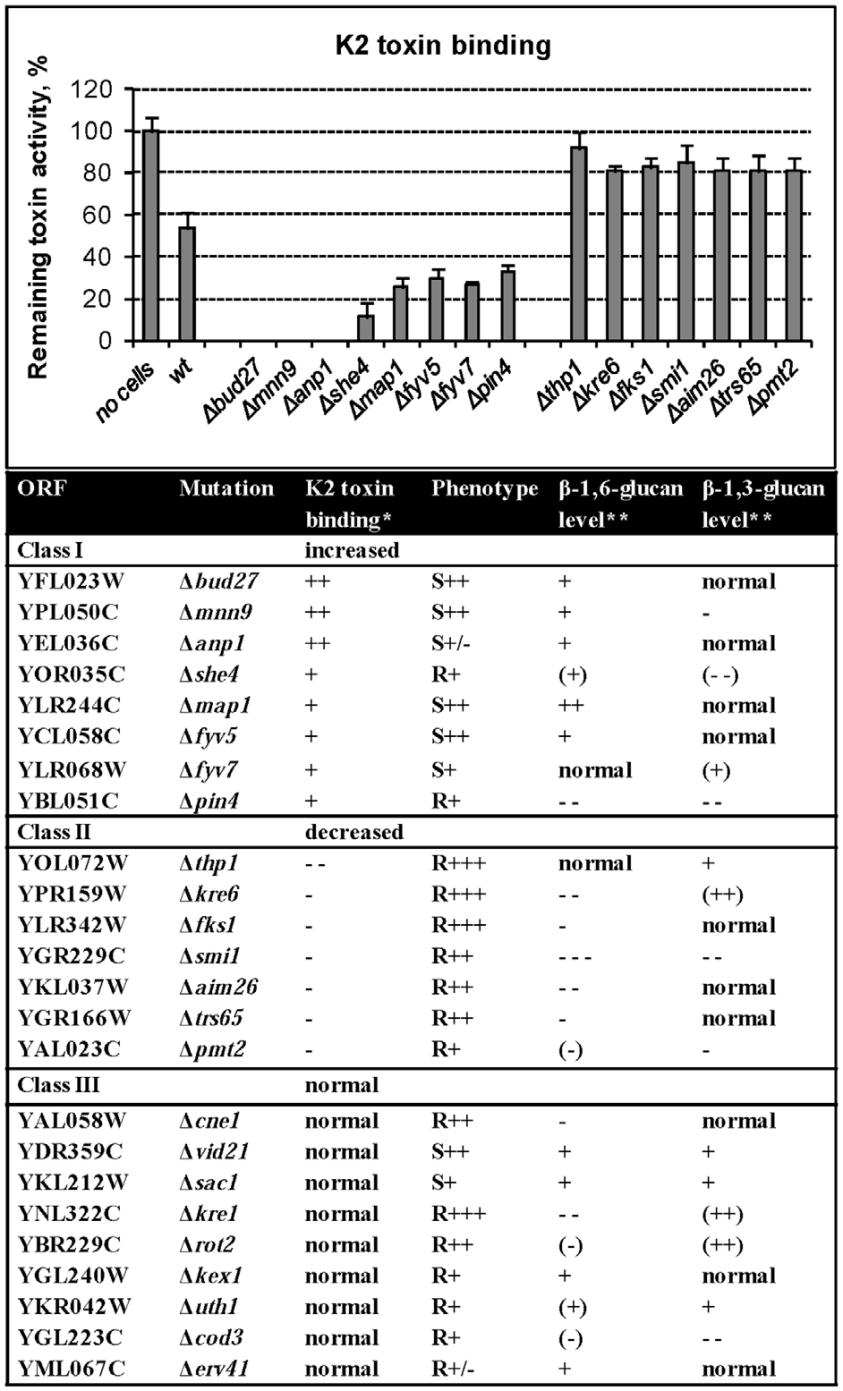
K2 toxin binding properties of mutants with altered K2 resistance. K2 toxin binding assay, as described in [28]. The histograms depict the remaining K2 toxin activity following incubation of K2 toxin preparations either with wild-type reference cells or with the mutant cells to be tested. This experiment was repeated three times. Error bars indicate the standard error of the mean. The effects on remaining toxin activity were all significant (p value <0.05). The remaining toxin activity is provided as a percentage of the activity of mock-treated K2 toxin preparations (see Materials and Methods). The table is an attempt to correlate K2 toxin binding with the resistance/sensitivity phenotype and the level of β-glucan. Toxin binding (*) was calculated as a percentage of the remaining toxin activity. Incubation with wild-type cells led to 45–65% of remaining activity;**++**corresponds to 0–10%,**+**to 10–40%; **−** to 70–90% and **–** to 90–100%. K2 resistance phenotypes are taken from Tables S1 and S2. The level of β-glucan (**) in these strains was previously published [27], and is as follows. Increase (I) of β-glucan: ++, 65<I<100; +, 45<I<65; (+), 25<I<45; (++), I<25%. Decrease (D) of β-glucan: − − −, 85<D<100; − −, 65<D<85; −, 45<D<65; (−), 25<D<45; (− −), D<25%.

As an initial step towards characterizing the effects of mutations causing altered sensitivity/resistance to K2 toxin, and as a mean to further validate our identification of novel K2 effectors, we characterized the K2 toxin binding property of a subset of 24 yeast mutant strains, using a previously established assay [28]. These mutants were selected because of their characterized level of β-glucan [27], which is strongly suspected to act as primary cell wall receptor for K1 and K2 toxins. The toxin binding assay simply consisted in measuring the remaining killing activity of K2 toxin preparations, following incubation either with reference wild-type cells or with the mutant to test (Figure 5, see also [28] and Material and Methods). Mock-treated K2 toxin preparations provided readouts of the maximal level of toxin activity, which was reduced by at least 2-fold upon incubation with wild-type cells (Figure 5). The killing activity was substantially increased upon incubation with cells lacking any of 7 proteins (Kre6, Fks1, Thp1, Smi1, Aim26, Trs65, and Pmt2). This indicates that these proteins are normally required for efficient K2 toxin binding. Note that Δ*kre6* cells were previously shown to be defective for toxin binding and therefore provide an internal control in our assay [26]. Conversely, the residual toxin activity was significantly reduced upon incubation with cells deleted for any of 5 genes (*SHE4, MAP1, FYV5, FYV7* and *PIN4*), and virtually abolished upon incubation with cells carrying a deletion in any of 3 genes (*BUD27, MNN9* and *ANP1*). This indicates that in the absence of any of these 8 gene products the binding of the toxin is favored. Finally, the individual inactivation of 9 genes (*VID21*, *SAC1*, *KRE1*, *ROT2*, *KEX1*, *UTH1*, *COD3*, *ERV41*, and *CNE1*) had no significant effect on toxin binding despite the fact that it conferred altered resistance/sensitivity toward the toxin (see Table in Figure 5).

We observed a good correlation between the genes whose deletion led to decreased toxin binding and increased resistance to the toxin (Figure 5, Class II). Similarly, many genes whose deletion led to increased toxin binding also correlated quite well with increased sensitivity towards the toxin (Figure 5, Class I). Finally, that the biology of K2 toxin, i.e. its modes of interaction with the host cell and its killing action, is highly complex and cannot simply be recapitulated by a single step of toxin binding to the cell is illustrated by the observation that deletion of genes that had no effect on toxin binding led to both increased resistance and increased sensitivity.

In an attempt to correlate toxin binding with the sensitivity/resistance towards the toxin and with the composition of the cell wall, we further addressed the respective levels of β-1,3- and β-1,6-glucan in the mutants described in [27]. We observed no correlation between K2 toxin binding, the resistance phenotype, and the level of β-1,3-glucan (Figure 5). On the other hand, the level of β-1-6-glucan was often decreased in resistant cells that also showed reduced toxin binding, and reciprocally, β-1-6-glucan level was often increased in sensitive cells that also showed increased toxin binding.

We concluded that there is a good correlation between the sensitivity/resistance towards K2 and the ability of the cells to bind the toxin. In addition, our observations are compatible with a model in which mutants with deficiencies in cell wall β-1,6-glucan are more likely to bind less effectively the toxin and therefore to be resistant, and *vice versa*. β-1,6-glucan thus appears to play an important role in K2 toxin binding. This is consistent with the results obtained with the *kre* mutants, which contain decreased level of β-1,6-glucan and were shown to be resistant to K1 toxin [16]. However, at this stage this is strictly indicative owing to the limited set (24 strains) tested in our binding assay.

## Discussion

### Probing the Yeast Genome for Novel K2 Toxin Effectors

**Figure 6:**
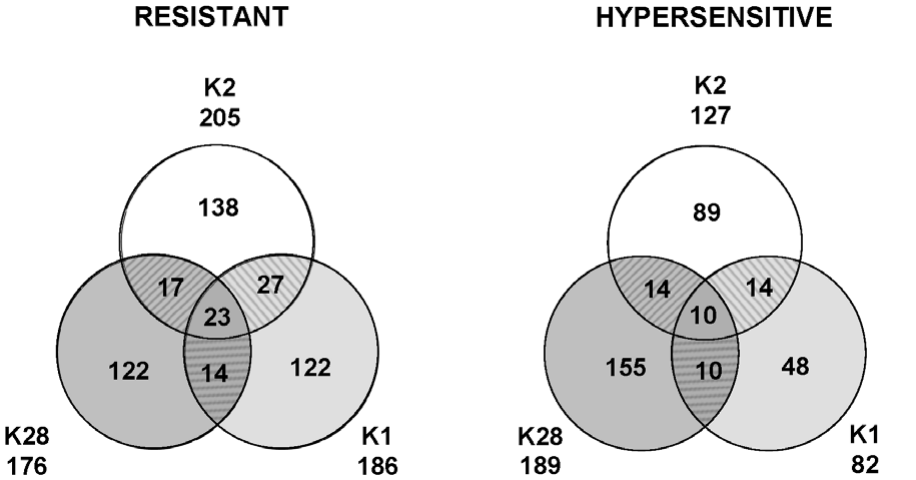
Genes involved in K2 biology are highly specific. The Venn diagrams depict the number of gene products known to contribute to the phenotypes of the three major killer K toxins. The number of mutants, involved in K2 resistance/hypersensitivity identified in our screens, is a combination of those exhibiting “strong” and “weak” phenotypes. The number of genes, affecting K1 and K28 biology are presented according to references [27] and [28], respectively.

When we initiated this work, genome-wide screens had been performed in budding yeast to identify genes involved in controlling the sensitivity/resistance towards K1 and K28 toxins [27], [28]. Similar studies had not been performed for the killer K2 toxin. Around 300–400 gene products had been identified for each toxin tested (268 for K1 and 365 for K28). Out of these, only very few are involved in the biology of both K1 and K28 (Figure 6). This is in line with the conclusion that K1 and K28 toxins have distinctly different modes of action, either disrupting cell integrity at the cell wall/plasma membrane (for K1), or affecting host cell gene expression in the cell nucleus and triggering cell cycle defects and apoptosis (for K28) [11], [14]. K1 and K2 toxins are both acting primarily at the cell wall/plasma membrane. However, some evidences suggested that these two toxins might function differently (see Introduction), prompting us to conduct genome-wide screens aimed at identifying novel K2 toxin effectors. We tested the yeast knock out collection, comprised of ∼5000 individual strains deleted for a single nonessential gene, and scored for any growth defect or growth enhancement (Figure 1A). After retesting the initial candidates by three independent assays in triplicate (Figure 1B), we identified 332 host factors involved in K2 resistance or hypersensitivity.

A major conclusion from this work is that the vast majority of the genes identified in our K2 screens were not previously characterized in the biology of K1 or K28 toxins (see Figure 6 for a summary and Figure S3). This indicates that each of the three major K toxins kill cells according to a specific *modus operandi*. Another important conclusion is that the genes involved in increased resistance towards K2 toxin are strikingly involved in different biological processes, or encode different cellular components, than those involved in hypersensitivity (Figures 2 and 3, see also Tables S1, S2, S3). Finally, the gene products involved in increased resistance and hypersensitivity are highly interconnected within functional and physical networks in the host cell (Figure 4 and Figure S2).

### K2 Resistance and the Cell Wall Structure

**Figure 7:**
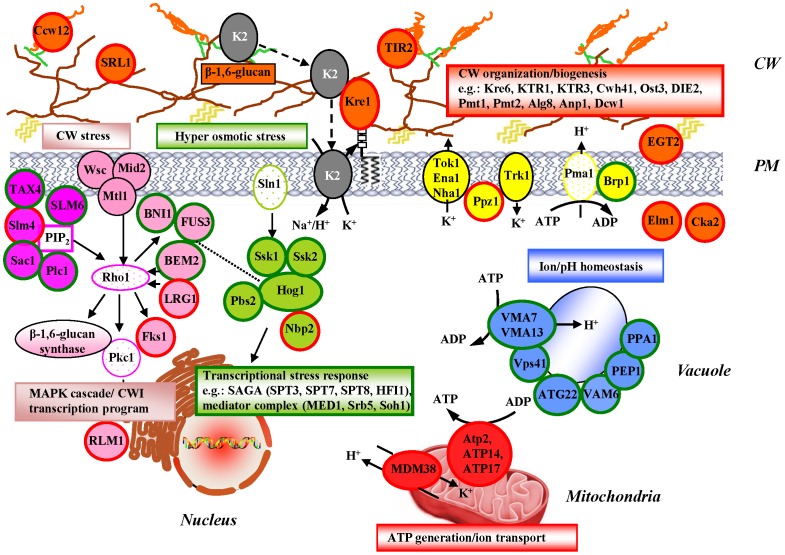
Model for K2 toxin entry and response of the host cell. The K2 toxin (grey oval) initially binds to cell wall-localized β-1,6-glucan. K2 is recognized by the plasma membrane-localized receptor Kre1 prior to integrating into the cell membrane and disrupting the electrochemical ion gradient leading to the leakage of K^+^ ions. Disrupting the functional integrity of the plasma membrane triggers multiple stress signaling responses involving the cell wall integrity pathway (in pink), the HOG pathway (in green), and phosphoinositide signaling (in purple). Rho1 and Pkc1 efficiently relay cell wall stress from the plasma membrane into the nucleus, leading to the activation of specific transcriptional programs. Ion and pH homeostasis maintenance mechanisms are activated to prevent cell death through ion leakage. Plasma membrane ion channels and their regulators (in yellow), vacuolar H^+^-ATPase and other vacuolar ion homeostasis keeping proteins (in blue), mitochondrial F0F1 ATP-synthase and H^+^/K^+^-antiporter (in red) are all involved. Key: Gene products depicted in uppercase denote K toxin effectors which are specific to K2 toxin. Lowercase genes are also involved in K1 and/or K28 susceptibility. Gene products circled in red are involved in K2 resistance; those circled in green in K2 hypersensitivity. For the K2 effectors, the color-coding is as follows: Orange, proteins associated with cell wall organization/biogenesis; Purple, phosphoinositide-related; Pink, CWI cascade; Green, HOG pathway; Red, mitochondrial constituents; Blue, vacuolar constituents; Yellow, ion transporter at the PM. CW, cell wall; PM, plasma membrane; CWI, cell wall integrity pathway. The cell wall consists of an inner layer, which is composed of β-1,3-glucan (in brown) and chitin (yellow waves), and an outer layer, densely packed with mannoproteins (in orange), extensively modified by *N*- and *O*-glycosylation (not represented). Components of the inner and outer cell wall layers are connected by β-1,6-glucan (in green).

Our screens identified gene products directly connected to cell wall organization and biogenesis as major determinants of resistance to K2 toxin. The yeast cell wall consists of an inner layer, which is composed of β-1,3-glucan and chitin, and an outer layer, densely packed with modified mannoproteins (see Figure 7 and [29]). Components of the inner and outer cell wall layers are connected by β-1,6-glucan. Defects in mannosylation and in β-1,6-glucan synthesis are known to lead to killer K toxin resistance, suggesting that these components are directly required for the formation of functional toxin receptors [1], [27]. Consistently, we identified several mutations in genes encoding proteins affecting β-glucan synthesis and assembly (Kre6, Fks1, Cwh41), involved in *N*- and *O*-linked protein glycosylation (Ost3, Pmt1, Pmt2, Alg8, Anp1), and connected to membrane organization and function (Drs2, Lem3), as conferring increased resistance to K2 toxin (Figure 7, circled in red). These genes were also identified in K1 genome-wide screens, suggesting that they are important for cell wall and plasma membrane receptor formation for both killer toxins.

In addition, we found several genes involved in K2 resistance, which importantly were not identified in K1 screens (in uppercase in Figure 7, and see Figure S3). These encode the mannoproteins Srl1, Tir2 and Ccw12, the established and putative mannosyltransferases Ktr1 and Ktr3, respectively, and the putative mannosidase Dcw1. This highlights that mannosylation is likely a key specific determinant in the formation of functional K2 receptors. Note that both Ccw12 and Dcw1 were also identified as effectors of K28 resistance [28] (see Figure S3 and Table S2).

Our dataset on K2 toxin is compatible with the earlier proposal for K1 that β-1,6-glucan is a major contributor to cell wall receptors (Figure 5). Defects in β-1,6-glucan synthesis are notably caused by mutations in genes encoding proteins that function throughout the secretory pathway, consistent with the biosynthetic pathway of this polymer [27], [30]. We believe that this explains why we identified numerous ER-associated (e.g. Pmt1, Pmt2, Ost3, Rot2, and Alg8) and Golgi-localized (e.g. Fks1, Kex1, Kex2, and Drs2) proteins involved in carbohydrate metabolism as important modulators of K2 resistance. Additional ER-associated proteins (Cne1, Qri8, Sel1, and Scj1) are components monitoring the quality of the constituents of the receptors. Dysfunction of this protein folding surveillance pathway might lead to the accumulation of defective cell wall and/or plasma membrane receptors thereby affecting toxin recognition and/or binding.

Finally, we also identified several genes connected to the cell cycle, growth, and proliferation (e.g. *ELM1, CKA2, EGT2*) as modulators of K2 resistance, possibly reflecting the former conclusion that cell wall synthesis is tightly controlled and coordinated with the progression of the cell cycle, including the sequential use of specific cell wall proteins [31]. Of note, the Δ*elm1* and Δ*cka2* mutations cause resistance to all three K1, K2, and K28 toxins (see Figure S3), whereas the Δ*egt2* mutation, characterized by a cytokinesis defect [32], is strictly specific for K2.

### K2 Susceptibility and Cellular Stress Response Pathways

Cellular stress is known to elicit the activation of specific transcriptional circuitries such as the cell wall integrity (CWI), and the general stress response (HOG) pathways [33], [34]. The CWI pathway transmits cell wall stress signals from the cell surface via the plasma membrane-localized sensors Wsc’s and the mechanosensors Mid2 and Mtl1 to the Rho1 GTPase. Rho1 in turn mobilizes a physiological response through a variety of effectors, leading to substantial remodeling of the cell wall [33]. The subcellular localization of Rho1 and its activity are regulated by a set of GTPase activating proteins, including Lrg1, Bem2, Sac7, and Bag7, each signaling in specific regulatory loops. We found that the inactivation of *LRG1* led to increased K2 resistance. This is probably due to decreased Fks1 activity and subsequent alteration in cell wall structure. We also noted that deletion of *BEM2* caused hypersensitivity to K2 toxin, possibly because of defects in the cytoskeleton organization involving the formin Bni1. Consistently, deletion of *BNI1* or deletion of its activator *FUS3* also caused K2 hypersensitivity. Deletion of *FUS3* led to an even higher level of hypersensitivity to K2 toxin compared to the single Δ*bem2* or Δ*bni1* mutations, probably because of its possible connection with the HOG signaling pathway (see dashed line in Figure 7). Cell wall damage is further signaled from Rho1 through a Pkc1-controlled MAP kinase cascade to the transcription factors Rlm1 and SBF [33]. Deletion of *RLM1*, whose gene product controls the expression of the majority of the genes involved in cell wall biogenesis, unsurprisingly led to K2 resistance. Taken together, we found five unique mutations Δ*lrg1,* Δ*bem2*, Δ*bni1*, Δ*fus3*, and Δ*rlm1*, not detected in the K1 or K28 genome-wide screens (see Figure S3, Tables S1 and S2), unequivocally demonstrating the crucial importance of the CWI pathway in the specific response to K2 toxin-induced stress.

Phosphoinositides (PIs) play an important role in both Rho1 activation, and in the recruitment of some of its effectors to the plasma membrane [33]. Surprisingly, we found that the deletion of *PLC1* or *SAC1*, involved in the synthesis of PIs, led to K2 hypersensitivity. This is in contrary to the strong resistance to K1 toxin caused by such deletions (see Figure S3, Tables S1 and S2). We also observed that the deletion of *TAX4*, involved in the regulation of PI(4,5)P_2_ level, caused hypersensitivity, whereas the sensitivity to K1 toxin in such mutant is unaltered [27] (see Figure S3). In addition, we noted alterations in K2 sensitivity when *SLM4* or *SLM6* were deleted. For SLM4, this was also observed for toxin K28 but not for K1 (see Figure S3). *SLM4* and *SLM6* exhibit synthetic genetic interactions with *MSS4* encoding an essential PI(4)P-kinase which produces the PI(4,5)P_2_ involved the Pkc1 pathway [35]. Our results thus demonstrate the importance of PI-signaling in triggering the K2 toxin-mediated CWI surveillance pathway.

The HOG (*h*igh *o*smolarity *g*lycerol) signaling pathway is also activated when cells undergo stress provoked by ion loss such as following the action of K2 toxin. Hog1 is a mitogen-activated protein kinase, which is a major regulator of such stress response. Its upstream regulators include the MAP kinase Pbs2 and the two-component regulator Ssk1 and Ssk2 [34]. Our observation that the absence of Hog1, or of any of its three major regulators, led to K2 hypersensitivity, and the previous demonstration of similar effects for K1 and K28 toxins [27], [28] attest of the importance of the HOG pathway in response to the stress induced by all three toxins. This demonstrates a certain level of communalities between the three major K killer toxins.

Furthermore, we found that when the HOG pathway is constitutively induced, cells became more resistant to K2 toxin. This was observed in the absence of Nbp2, which normally negatively regulates Hog1 by recruiting the phosphatase Ptc1 to the Pbs2/Hog1 complex [36]. Finally, upon phosphorylation, Hog1 is translocated into the nucleus where it activates RNA polymerase II. We found that several RNA polymerase II ancillary factors, such as the SAGA transcriptional regulatory complex, are important for the cellular response to K2 action through its impact on transcription. Typically, their inactivation led to K2 hypersensitivity.

### Changes in K2 Resistance, Ion/pH Homeostasis, and ATP Generation

Yeast cells harbor ion channels, localized in several cellular compartments like the plasma membrane, the vacuole, and the mitochondria. These channels provide cells with ions, including potassium, which regulates the intracellular pH, maintains a positive turgor inside the cell, and helps to cope with osmotic stress. Cellular cation homeostasis is maintained on one hand, by the K^+^ and by the Na^+^/K^+^ transporters Nha1, Tok1, and Ena1, which mediate the efflux of the K^+^ ions out of the cell, and on the other hand, by the Trk1, Trk2 and Nsc1 K^+^ transporters, involved in the influx of K^+^ [37]. Besides its other numerous cellular functions, the HOG signaling pathway is also involved in the elimination of K^+^ ions from the cell through the induction of a transcriptional response, regulating the production of the Nha1 and Ena1 channels. Another regulator, the Ppz1 phosphatase, also modulates the expression of *ENA1* gene and the activity of Trk1 [38]. We observed that deletion of *PPZ1* results in K2 toxin resistance, probably due to an elevated turgor pressure following the increased intracellular concentration of potassium and the constitutive activation of Mpk1 [33]. The deletion of *PPZ1* also led to increased resistance to toxin K28 but not to K1 (see Figure S3).

In addition, cytosolic pH homeostasis is regulated by several plasma membrane and vacuolar H^+^-ATPases [37]. One of these is the H^+^-ATPase Pma1, which is essential for viability. We observed that the deletion of *BRP1*, which is known to downregulate the expression of *PMA1,* leading to a decreased H^+^ efflux from the cell, resulted in hypersensitivity to K2 toxin. This is exactly opposite to what was reported for K1 where deletion of *BRP1* led to toxin resistance [27] (see Figure S3). Also, mutations in genes encoding the vacuolar H^+^-ATPase subunits Vma7 and Vma13 led to the perturbation of pH homeostasis due to defective proton transport into the vacuole, resulting in a hypersensitivity to K2 toxin. None of the *Δppz1*, *Δvma7*, and *Δvma13* mutations affected K1 resistance [27] (see Figure S3, Tables S1 and S2), suggesting that the efficiency of the cellular response to the action of this toxin is less sensitive to alterations in intracellular pH. In addition to deletion of genes encoding the vacuolar H^+^-ATPase subunits, we observed that deletion of numerous other genes encoding vacuolar components (e.g. *VAM6*, *VPS41*, *PEP1*) led to K2 hypersensitivity likely due to defects in the osmoregulation and ion homeostasis maintenance machinery. Ion and pH homeostasis therefore appear of paramount importance in controlling the cellular response to the action of K toxins.

Finally, deletion of numerous genes encoding mitochondrial ribosomal proteins, constituents of the mitochondrial electron transport chain, and both structural and regulatory subunits of the F1F0-ATP synthase, led to increased resistance to the K2 toxin. We speculate that this is due to decreased cellular ATP level, which is required for the activity of cation channels such as Ena1 to Ena5 [37] and possibly for the activity of new channels induced by K2 toxin.

### Conclusions

In summary, we identified 332 novel K2 effectors and demonstrated that K2 toxin resistance and hypersensitivity largely map in distinct cellular pathways: cell wall/plasma membrane structure and biogenesis, and the respiratory function for K2 resistance, and stress-signaling and ion and pH homeostasis for hypersensitivity. Notably, we report that only a minority of factors identified in our work is involved in the susceptibility to all three major K toxins: K1, K2, and K28. This was expected for K28, which is mostly active in the nucleus, but much less so for K1 and K2, which are both primarily active at the cell surface. We further show that several host factors have opposite effects on K1 and K2 phenotypes. While we can not formally exclude the possibility that several host factors might have escaped previous detection in the K1 and K28 genome-wide screens, simply because they might impact differently K toxin biology under the specific assay conditions used in these screens (such as temperature, pH, medium or the tester strain used), the vast majority of the host factors identified in our work had not been linked to K toxin biology before. We would like to suggest that during evolution yeast cells acquired a specific K2 toxin effectors set.

## Materials and Methods

### Strains and Media

Experiments were performed with a collection of *Saccharomyces cerevisiae* strains (BY4741 background, *MAT*a; *his3*Δ*1*; *leu2*Δ*0*; *met15*Δ*0*; *ura3*Δ*0*), where single ORFs, identified in this organism, were replaced by KanMX4 module (4784 strains in total). This collection was kindly provided by Prof. Boone (Univ. of Toronto, Canada). Additional strains were purchased from Thermo Scientific Molecular Biology (Lafayette, CO, USA). For screening the YKO library, the K2 toxin-producing strain M437 (*wt, HM/HM [kil-K2]*) was used. To directly compare the resistance phenotypes of yeast mutants towards the K1, K2, and K28 toxins, the additional yeast strains K7 (*MATa arg9 [kil-K1])* and MS300 (*MATα leu2 ura3-52 [kil-K28]*) were used.

Yeast strains were grown in standard YPD medium (1% yeast extract, 2% peptone, 2% dextrose, 2% agar). For screening purposes, MBA medium (0.5% yeast extract, 0.5% peptone, 2% dextrose), adjusted to pH 4 with the 75 mM phosphate-citrate buffer, supplemented with 0.002% methylene blue dye, was used. To allow a direct comparison of the K1, K2 and K28 killer phenotypes (see Figure S3), the pH of the MBA medium was adjusted to 4.8.

### Genome-wide Screening Procedure

Library of deletion mutants was screened by performing 4 individual primary and 3 secondary screens. In the primary screens, the collection of mutants, arrayed in 96 colony format were inoculated either onto the YPD-agar or liquid YPD and grown overnight at 30°C. Afterwards, cells were transferred onto the MBA plates that had been seeded with an overlay of the overnight pre-culture K2 toxin producing strain (2×10^5^ cells/plate (primary screen No. 1) or 2×10^6^ cells/plate (No. 2)) or purified K2 toxin (300 U/plate (No. 3) or 600 U/plate (No. 4)). K2 toxin was isolated as described previously [22], and the activity was determined as in [39]. MBA plates that did not contain either K2 producing strain or K2 toxin were used as control for the growth of the yeast library. After 2 days of incubation at 25°C, potential resistant or hypersensitive candidates, differing in size and/or color from their neighbors and corresponding controls and exhibiting strong phenotypes (R++/S++, the scoring categories are described in Tables S1 and S2) in at least one of the screening conditions (using K2 producing strain or K2 toxin), were selected and re-tested by performing three secondary screens. The sensitivity was tested by either deposing 100 µL (∼10 U) of purified K2 toxin into 10 mm diameter “punched-wells” in the agar plate (secondary screen, No. 5), or spotting either 5 µL (∼0.5 U) of toxin or overnight pre-cultured and concentrated K2-producing cells onto the MBA medium (secondary screens, No. 6 and No. 7). In all 3 cases, the MBA was overlaid with the yeast strain of interest (2×10^6^ cells/plate). Strains were grown for 2 days at 25°C, and the diameter of the lysis zones measured. The secondary screens (No. 5–7) were each repeated 3 times. For the quantitative data, the values obtained in screen No. 5 were used. A t-test was used to evaluate the phenotypical significance of the differences between the wild type and mutant strains. The differences were considered significant for p<0.05. Finally, ∼25% of the 332 candidate mutants identified in our screens were shown by diagnostic PCR to carry the expected deletion (see Tables S1 and S2).

### Toxin-cell Binding Assay

To assess binding of K2 toxin to the yeast, 10 OD of wild-type (BY4741) or mutant cells grown overnight at 30°C in YPD were incubated in 1 ml (about 100 U) of K2-containing supernatant for 15 minutes at 4°C. Then, the cells were removed by centrifugation (10000×g, 5 min), and the remaining supernatant was tested for activity by using the well assay [39]. Binding level was expressed as the percentage of toxin activity obtained for each mutant compared to the one of the non-incubated with the cells K2 toxin.

### Bioinformatics

#### GO terms enrichment

Statistical validation of cellular pathways related to K2 function: processes, functions, and components annotated to the set of K2 resistant or hypersensitive genes (Table S1) were compared to a background set of genes, for which the scores were generated according to the method described in [40]. Data were generated using the BiNGO 2.44 plug-in embedded into the Cytoscape 2.8.2 platform. Significance *P* values were calculated with the hypergeometric test, using the Benjamini and Hochberg false discovery rate (FDR) correction for the enrichment of each GO term. Fold enrichment (F.E.) was determined by dividing the frequency of specific gene cluster to the total frequency for each GO term.

#### Network construction

Network diagrams were generated using STRING web resource (http://string-db.org,) [41]. Our created network uses the “confidence view” option of the program, where stronger associations are represented by thicker lines. The experiments-based active prediction method was used, and the medium confidence score (0.400) was utilized. To simplify the presentation, network was also redrawn manually by selecting the genes involved in selected biological processes annotated by the GO program.

#### Venn diagrams

They were created manually, comparing our data of the K2 screen with the ones obtained in K1 [27] and K28 [28] screens.

## Supporting Information

Figure S1 **Scoring categories of the K2 mutants identified in our screens.** In screens No. 1 and 2 (see Figure 1), the scoring categories of K2 mutants were based both on the color (ranging from white, for very resistant mutants, to intense cyan, for very sensitive ones) and the size of the colonies (from very large, for very resistant mutants, to very small, for very sensitive ones). In the resistant (R) category, we referred to: R+ (light blue), R++ (white), R+++ (white and big colonies), whereas in the sensitive (S) category we referred to: S+ (blue), S++ (deep blue), S+++ (cyan and very small colonies). BY4741 cells consistently showed bluish colony color in our assay. In screens No. 3 and 4, the scoring categories were only based on the size of the colonies relative to wild-type (wt). Sensitive mutants (S) were selected on plates containing low concentrations of toxin (300 U/plate): S+ (small colonies), S++ (trace of colonies), S+++ (no colonies). Mutants unaffected for K2 sensitivity showed colonies similar in size to wild-type (wt). Resistant mutants (R) were selected on plates containing high concentrations of toxin (600 U/plate): R+ (small colonies), R++ (average-sized colonies), R+++ (large colonies). Mutants unaffected for K2 resistance consistently showed no growth, similarly to the wt control. Scoring categories of screens No. 5, 6 and 7 are based on the size of the “halo” respective to that obtained with wild-type cells. The wild-type control cells (BY4741) consistently showed a 2.5–3 mm radius “halo” in our assay. Resistant strains showed smaller “hallo”; sensitive strains “larger” ones. The categories were defined as such: R+ (1–2 mm radius), R++ (0.5–1 mm radius), R+++ (0.5–0 mm radius); S+ (3.5–4 mm radius), S++ (4–5 mm radius), S+++ (over 5 mm radius). Finally, it should be noted that in screens 1–4, the evaluation was strictly visual and the scores arbitrary, while in screens 5–7, the size of the “halo” was measured.
(TIF)

Figure S2 **Physico-functional networks of K2 effectors identified in this work.** The networks were established with STRING, see Materials & Methods.
(TIF)

Figure S3 **Representative examples of gene products affecting differentially the susceptibility towards the three major killer toxins.** The assay is the same as the one used in screen No. 7 (see legend to Figure 1 for details). K1, K2, or K28 toxins producing cells were deposited on the surface of an agar plate inoculated with a mutant strain to test. In this assay, a pH of 4.8, at which all three toxins are at least partially active, was used. Scoring categories are based on the size of the “hallo”/lysis zones: R - resistant, S - sensitive, wt - comparable to BY4741. In each case, a representative image, obtained with one of the mutant listed in each category, is shown.
(TIF)

Table S1 Mutant strains with an altered K2 killer toxin phenotype.
(XLS)

Table S2 Mutant strains with weak altered K2 killer toxin phenotype.
(XLS)

Table S3 Gene Ontology terms for K2 resistant and hypersensitive genes.
(XLS)
