# Supplementary figures and images for: Screening the Budding Yeast Genome Reveals Unique Factors Affecting K2 Toxin Susceptibility
Source: PLoS One. 2012 Dec 5;7(12):e50779. doi: 10.1371/journal.pone.0050779 (PMC3515549; doi:10.1371/journal.pone.0050779)

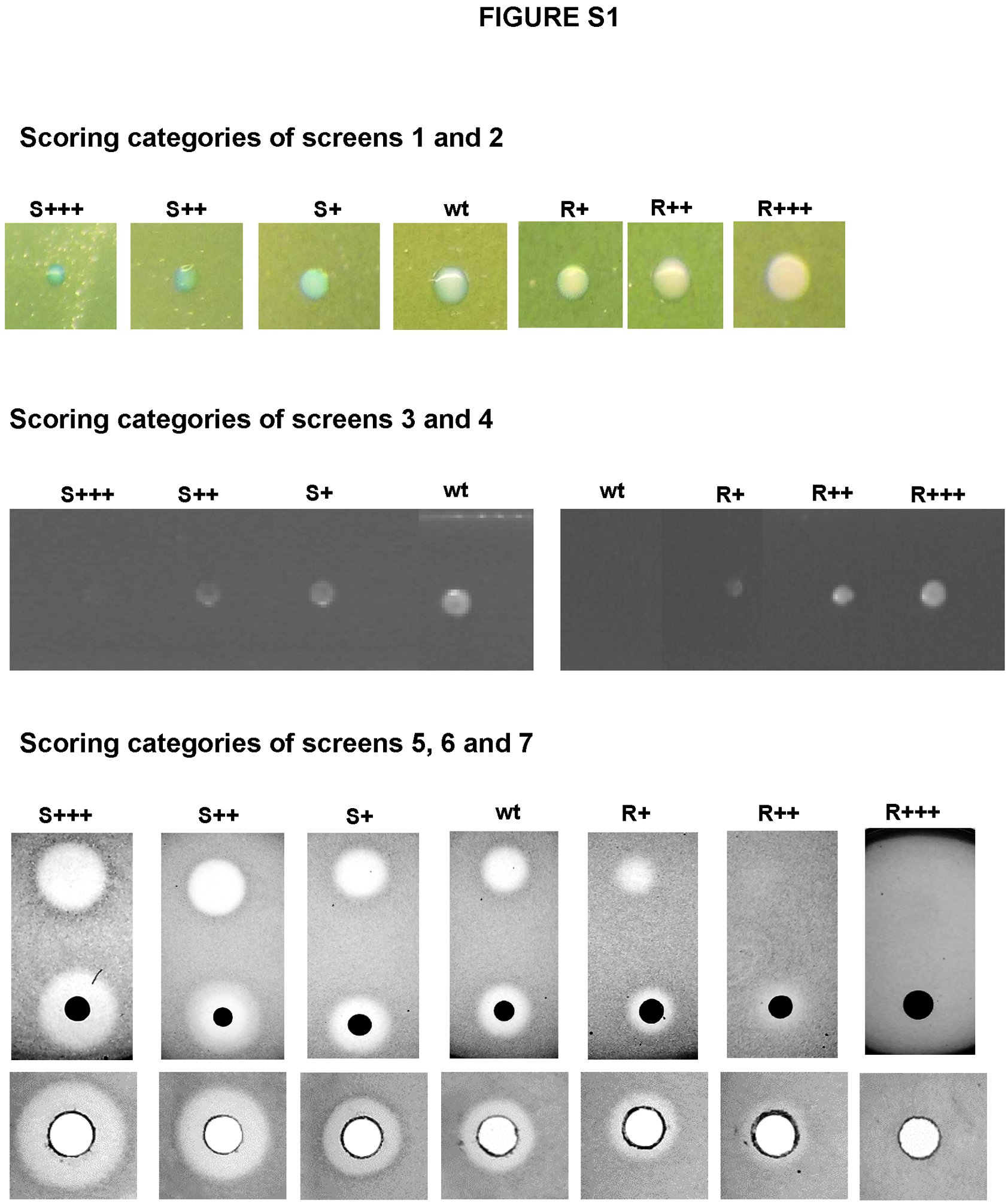

Supplement: Figure S1 — Scoring categories of the K2 mutants identified in our screens. In screens No. 1 and 2 (see Figure 1), the scoring categories of K2 mutants were based both on the color (ranging from white, for very resistant mutants, to intense cyan, for very sensitive ones) and the size of the colonies (from very large, for very resistant mutants, to very small, for very sensitive ones). In the resistant (R) category, we referred to: R+ (light blue), R++ (white), R+++ (white and big colonies), whereas in the sensitive (S) category we referred to: S+ (blue), S++ (deep blue), S+++ (cyan and very small colonies). BY4741 cells consistently showed bluish colony color in our assay. In screens No. 3 and 4, the scoring categories were only based on the size of the colonies relative to wild-type (wt). Sensitive mutants (S) were selected on plates containing low concentrations of toxin (300 U/plate): S+ (small colonies), S++ (trace of colonies), S+++ (no colonies). Mutants unaffected for K2 sensitivity showed colonies similar in size to wild-type (wt). Resistant mutants (R) were selected on plates containing high concentrations of toxin (600 U/plate): R+ (small colonies), R++ (average-sized colonies), R+++ (large colonies). Mutants unaffected for K2 resistance consistently showed no growth, similarly to the wt control. Scoring categories of screens No. 5, 6 and 7 are based on the size of the “halo” respective to that obtained with wild-type cells. The wild-type control cells (BY4741) consistently showed a 2.5–3 mm radius “halo” in our assay. Resistant strains showed smaller “hallo”; sensitive strains “larger” ones. The categories were defined as such: R+ (1–2 mm radius), R++ (0.5–1 mm radius), R+++ (0.5–0 mm radius); S+ (3.5–4 mm radius), S++ (4–5 mm radius), S+++ (over 5 mm radius). Finally, it should be noted that in screens 1–4, the evaluation was strictly visual and the scores arbitrary, while in screens 5–7, the size of the “halo” was measured. (TIF) [file pone.0050779.s001.tif]

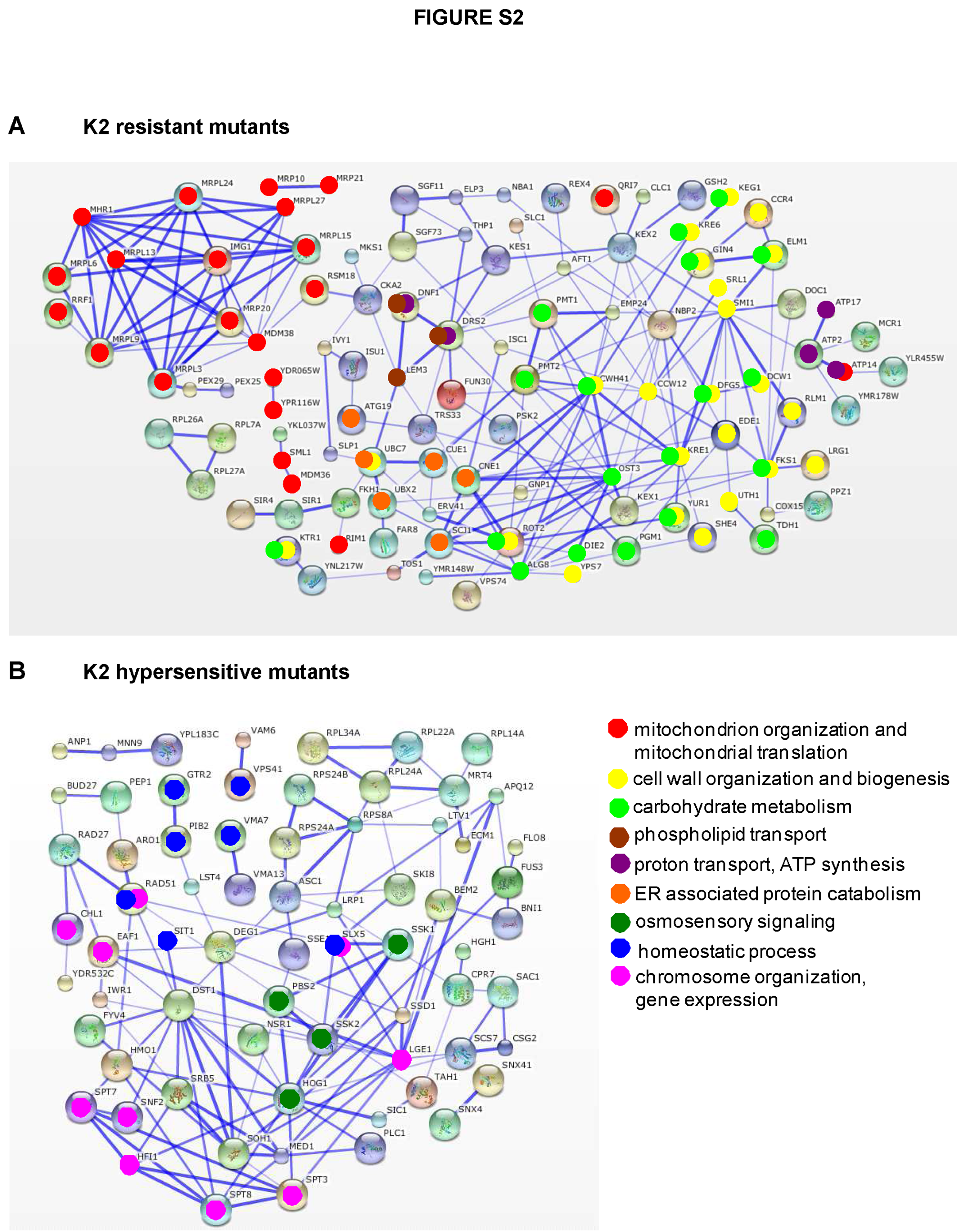

Supplement: Figure S2 — Physico-functional networks of K2 effectors identified in this work. The networks were established with STRING, see Materials & Methods. (TIF) [file pone.0050779.s002.tif]

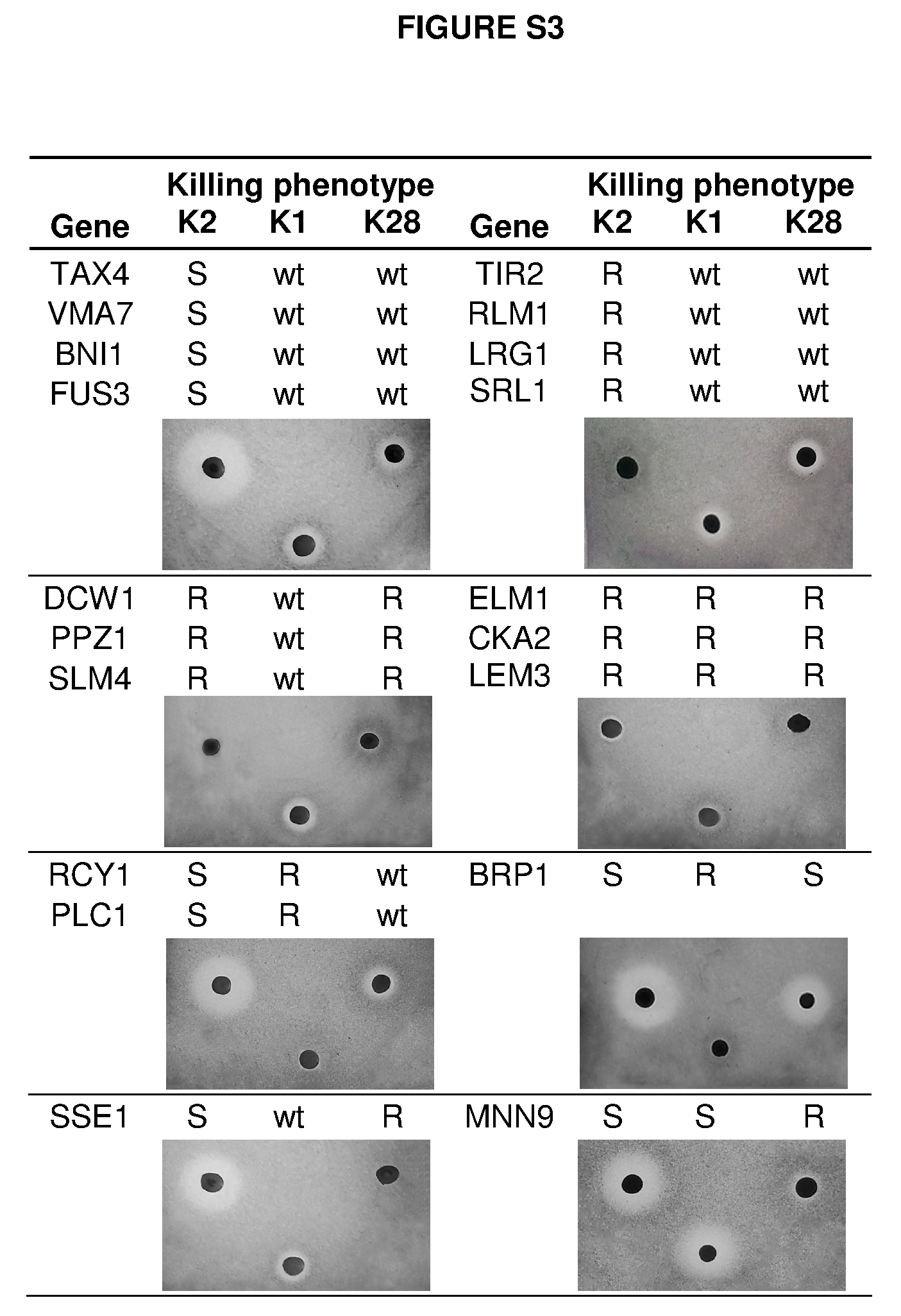

Supplement: Figure S3 — Representative examples of gene products affecting differentially the susceptibility towards the three major killer toxins. The assay is the same as the one used in screen No. 7 (see legend to Figure 1 for details). K1, K2, or K28 toxins producing cells were deposited on the surface of an agar plate inoculated with a mutant strain to test. In this assay, a pH of 4.8, at which all three toxins are at least partially active, was used. Scoring categories are based on the size of the “hallo”/lysis zones: R - resistant, S - sensitive, wt - comparable to BY4741. In each case, a representative image, obtained with one of the mutant listed in each category, is shown. (TIF) [file pone.0050779.s003.tif]
